# Establishing a mental lexicon with cochlear implants: an ERP study with young children

**DOI:** 10.1038/s41598-017-18852-3

**Published:** 2018-01-17

**Authors:** Niki K. Vavatzanidis, Dirk Mürbe, Angela D. Friederici, Anja Hahne

**Affiliations:** 1 Max Planck Institute for Human and Cognitive Brain Sciences, Leipzig, Germany; 2 Saxonian Cochlear Implant Center, Technische Universität Dresden, Dresden, Germany

## Abstract

In the present study we explore the implications of acquiring language when relying mainly or exclusively on input from a cochlear implant (CI), a device providing auditory input to otherwise deaf individuals. We focus on the time course of semantic learning in children within the second year of implant use; a period that equals the auditory age of normal hearing children during which vocabulary emerges and extends dramatically. 32 young bilaterally implanted children saw pictures paired with either matching or non-matching auditory words. Their electroencephalographic responses were recorded after 12, 18 and 24 months of implant use, revealing a large dichotomy: Some children failed to show semantic processing throughout their second year of CI use, which fell in line with their poor language outcomes. The majority of children, though, demonstrated semantic processing in form of the so-called N400 effect already after 12 months of implant use, even when their language experience relied exclusively on the implant. This is slightly earlier than observed for normal hearing children of the same auditory age, suggesting that more mature cognitive faculties at the beginning of language acquisition lead to faster semantic learning.

## Introduction

### Cochlear implantation in profoundly hearing impaired and deaf children

In children with congenital or acquired sensorineural deafness the inner ear hair cells do not transform the mechanical sound waves into electrochemical signals, and thus the auditory nerve and consequently the auditory cortex remain unstimulated. They can nevertheless experience auditory input if provided with a cochlear implant (CI), a neuroprosthetical device that enables auditory input to the brain by directly stimulating the auditory nerve via electrical pulses. With children, one key motivation for such a hearing restoration is the parents’ wish for their children to be able to communicate orally, either because oral language is the family’s mode of communication, or because they want their children to participate in the mainstream oral world (including mainstream schools), or both. Consequently, language acquisition with the CI is a primary focus for these children, and yet we still know little about the actual stages and the time frame of language acquisition with an implant.

Hearing with an implant is different from natural hearing in several ways. First, despite the ongoing technological improvements, the CI provides only compromised dynamic range and frequency discrimination^1,2^. Hearing with a CI is thus more effortful^3^, especially in noisy environments^4^, and the actual benefit in understanding speech varies greatly, both for children and for adult users. Second, one has to consider, especially in the case of congenitally deaf children, that the maturing infant brain develops without any auditory input for a considerable amount of time. Even with an early implantation at about 12 months, implanted children will experience their first auditory input when normal hearing (NH) children have already explored language to an extent that allows them to produce their first words. We further know that a different set of available sensory modalities can influence the language network. Lane *et al*. found that congenitally blind individuals have a less left-lateralized language network, despite the available auditory modality^5^ and Finkl *et al*. (submitted) have shown that though the classical language network does not change with prelingual deafness, connectivity between speech-relevant areas are diminished. The exact implication of a delayed access to auditory input for children’s language acquisition remains to be elucidated.

### Language acquisition in normal hearing children

In order to better understand the demands of language acquisition with a CI, let us first consider the steps of language acquisition as they are presented by normal hearing children in the course of the first two years of their life. The chronological first two years are both a critical period in language acquisition and a time period considered as an early implantation age.

When NH children utter their first word at about 12 months, they have already accomplished a multitude of language specific perceptual steps. Already in utero they perceive the prosodic contours of their native language environment and imitate it in their crying as neonates^6^. Four months later, they show differences in the perceptual processing of native and non-native stress patterns^7^. Immersed in the continuous speech stream of their environment, they also begin to learn to recognize the phonemes of their native language, develop a preference for them^8^, and tune in to the specific phonotactics of their language, that is, the statistics of syllable and phoneme frequencies and distributions of the specific language^9^. These are not luxury accomplishments: Both stress pattern recognition and phonotactic rules are important heuristic cues for defining word boundaries in the ongoing speech stream^9–13^, as only after word boundaries have been established, can the child go on to recognize words and subsequently attach meaning to them. Recognizing words as familiar occurs around the age of 8 months^14^, though this does not yet mean that they have established a semantic relationship between the familiar word and the signified object. Friedrich & Friederici have shown that while infants are able to establish semantic relationships between single words and objects already by the age of 6 months, this effect is transitory and does not last until the next day^15^. It is not until the second year that robust semantic relationships are created between words and signified objects. A robust semantic relationship can be defined as not being arbitrary anymore, meaning that the misnaming of a known object will be perceived as a semantic violation. Such a semantic violation becomes evident in the electroencephalogram (EEG) at the age of 14 months, i.e. about the time of first word utterances^16^, and even at 12 months if a child is an early language learner^17^. For NH children, the second year of life is then also the most dynamic in vocabulary growth. While on average they start producing their first word around 12 months of age and learn about 50 words in the following six months, in the second half of their second year most children demonstrate a so-called vocabulary spurt in which they acquire new words at a tremendous speed^18,19^.

### Language acquisition with a cochlear implant

Clearly, a lot of ground is laid during the first year of life regarding the learning of auditory features of oral language. All this occurs even before producing the very first word and way before the understanding and production of new words gains momentum during the second year. An important question that thus arises for implanted children is how they master the steps of language acquisition if they have missed critical auditory input in these first years of development.

NH children demonstrate critical perceptual differentiation skills at birth for tone duration^20^, at 2 months for vowel length^21^, and at 4 months for stress pattern differentiation^7,22^. For implanted children, Vavatzanidis *et al*. were able to show that congenitally deaf children robustly differentiate between long and short vowel lengths after four months of implant use^23^ and, together with Segal *et al*., that implanted children differentiate between the (native) trochaic and the (non-native) iambic stress pattern after six months of implant use^24,25^. One can thus conclude that implanted children experience only a moderate delay until they master language relevant auditory differentiations. Having the resources to hear word boundary cues, however, does not have to automatically ensure further vocabulary acquisition. Thus, in the present study we address the next milestone in language acquisition, namely the formation of semantic relationships between words and objects.

### The N400 effect

To test for the formation of robust semantic relationships between words and objects in this study we employed the N400 effect as a neural marker. The N400 is an event-related potential (ERP) that reflects semantic processing. Whenever a semantic mismatch is encountered, for example when a picture is labelled incorrectly or a sentence finishes with a semantically incongruous word, this event-related potential has an increased negativity which is called the N400 effect^26,27^. The shape and latency of the N400 is modulated by a number of factors such as degree of violation (larger N400 effect for greater violations)^28,29^, age (ERP amplitude decreases and latency increases with age)^30^, and – critical for hearing impaired participants – listening effort (reduced N400 effect for degraded speech input)^31–33^. Most importantly for our purposes, the N400 effect marks receptive vocabulary formation since a semantic mismatch can only be perceived if there is an existing semantic relationship to be violated. It has thus a major advantage over behavioural studies: Whereas parental questionnaires are prone to parents’ bias and language assessment tests rely on the child’s cooperation and day form and require a certain minimum age, the ERPs directly reflect the underlying processes and they can be assessed for any age group.

To our knowledge, very few studies have attempted to establish the nature of the N400 in cochlear implanted adults or children and thus semantic processing with auditory input from a CI. Hahne *et al*. have investigated semantic and syntactic sentence processing in postlingually deafened adults with a CI. They found that CI users showed an increased latency and broader amplitude of the N400 effect than NH controls, attributed to the greater listening effort and the resulting higher cost of semantic integration^34^. The N400 effect can also be elicited in musical context, when a musical excerpt is followed by a word congruent or incongruent to features of the excerpt^35^. Bruns *et al*. could demonstrate that this effect is reproducible in postlingually deafened adults with a CI who had formed musical semantic concepts prior to their hearing loss. In contrast, prelingually deafened adults without such semantic concepts did not display an N400 effect^36^. Interestingly, the postlingually deafened CI users displayed an N400 effect of the same size and latency as NH controls, but made more errors behaviourally. Thus, the lack of confidence in their hearing judgement masked their actually good performance, which was only revealed by their NH equivalent ERP. In a study by Finke *et al*., an auditory oddball paradigm with words was employed, where CI users were asked to respond to the target words (living entities) and ignore the non-target words (non-living entities)^37^. This lexical rather than semantic mismatch task elicited an N400-like response at central and posterior sites, which was cautiously named N2/N4.

In the case of cochlear implanted children, only two studies have so far examined the nature of semantic processing with the N400 effect. Key *et al*. report a single case study where they find the N400 effect descriptively in a 6-year-old child, however, no statistical analysis was performed to confirm the effect^38^. Kallioinen *et al.* recently examined the semantic processing of 5- to 7- year-old children^39^. Fifteen children with an implant, fifteen children using a hearing aid (HA), and twelve NH controls participated in a semantic word-picture study where a word was presented acoustically followed by a picture presentation. There were two types of semantic mismatch presented: between-category (e.g. “wolf” – car) and within-category (e.g. “wolf” – bear) mismatches. All three participant groups displayed an N400 effect for both mismatch types – a frontocentral N400 for the within-category mismatch, and a centroparietal N400 for the between-category mismatch. Surprisingly, the CI group had the shortest latencies in both semantic mismatch types and the largest amplitude for the between-category mismatch, while in the HA and NH group it was the within-category mismatch that elicited the larger N400 effect. Note that in this study the CI group was heterogeneous with respect to hearing modality, with a large proportion of CI children using both an implant and a hearing aid. It would have been interesting to see the results of the bilaterally implanted children separately, as usually a hearing aid provides an advantage by having a wider dynamic range and better frequency discrimination than the CI. Also of interest would have been the separate analysis of the congenitally deaf or at least prelingually deafened children. Age at hearing loss varied greatly between 0 and 5 years, meaning that some implanted children were congenitally deaf and other children turned deaf after five years of normal maturation and thus after reaching the substantial language abilities of a 5-year-old.

The existing literature demonstrates that the violation of semantic relationships elicits the N400 effect in postlingually deafened adults and pre- and postlingually deafened school-aged children with a CI. However, no neural investigation of semantic relationship formation has been undertaken in implanted children during the critical second year of auditory input with the CI – a time frame equivalent to the second year of full language immersion in NH children, when vocabulary learning takes a leap. With the N400 effect as available marker, the present study investigates the development of word-object relationships over the course of one year, when the CI is the only source of auditory input.

We were specifically interested in the developmental trajectories of different performance groups as well as the development of the congenitally deaf children. We hypothesized to detect an N400 effect developing over the course of the second year as the children’s vocabulary emerges and grows, and to see the effect earlier in high performing children than in low performing children. We also expected to see a slower development of the N400 effect in congenitally deaf children as opposed to children with prior hearing experience as the latter had the advantage of at least some language immersion prior to cochlear implantation (ranging from actual word learning to at least access to phrasal boundaries and other prosodical cues).

## Methods

### Participants

Thirty-eight hearing impaired children with bilateral cochlear implants participated in the study. Of those, 6 had to be excluded from further analysis due to excessive artefacts. Of the remaining 32 children (14 girls), 13 had a severe or profound hearing loss with some residual hearing prior to implantation, which however was not sufficient for language acquisition despite the use of hearing aids. The other 19 children (8 girls) were diagnosed with congenital bilateral deafness. All children had underwent a pediatric audiological assessment consisting of a negative brain stem electric response audiometry (BERA), subjective audiometry, evaluation of specialized therapists on the child’s hearing reactions and speech development, and reports of the family. When a period of bilateral hearing aid use proved to be without benefit, cochlear implantation was performed on both ears (see details in Table 1).

**Table 1:** Details for implanted children entering the final analysis. Children with congenital deafness are marked with an asterisk.

	Sex	Aetiology	Bilateral Implantation Mode	Implant	Processor	Age @ activation in months
1	f	Neonatal jaundice	simultaneous	Concerto	Opus2	9
2*	m	Unknown	simultaneous	Sonata	Opus2	10
3*	m	Connexin-26 (c.35delG and c.139 G > T)	simultaneous	CI422	CP910	10
4*	f	Connexin-26 (c.35delG)	sequential	CI422	CP810	11
5*	m	Preterm birth (29 weeks gestational age)	sequential	CI422	CP810	11
6*	m	Connexin-26 (c.35delG and c.139 G > T)	simultaneous	CI422	CP810	11
7	f	Cytomegalovirus & Pendred-Syndrome	Simultaneous	Concerto	Opus2	11
8	m	Unknown	Simultaneous	CI422	CP910	11
9*	f	Unknown	Sequential	CI422	CP920	11
10*	f	Unknown	Sequential	Concerto	Opus2	12
11*	m	Unknown	Sequential	Concerto	Opus2	12
12*	m	Hereditary (MYO7A)	Simultaneous	HiRes90K Mid-Scala	Naida CI Q70	12
13	f	Unknown	Simultaneous	CI422	CP910	13
14*	m	Hereditary	Simultaneous	Concerto	Opus2	14
15*	f	Unknown	Simultaneous	CI512	CP810	16
16*	f	Hereditary	Simultaneous	CI512	CP810	16
17*	m	Hereditary	Simultaneous	CI24Re	Freedom	18
18*	m	Hereditary	Sequential	Concerto	Opus2	18
19*	m	Unknown	Simultaneous	CI512	CP810	20
20*	f	Unknown	Simultaneous	Concerto	Opus2	21
21*	m	Unknown	Simultaneous	CI512	CP810	22
22	m	Inner ear malformation	Simultaneous	Concerto	Opus2	23
23	m	Connexin-26 (c.35delG)	Sequential	Concerto	Opus2	25
24*	f	Hereditary	Simultaneous	Sonata	Opus2	26
25	m	Meningitis	Simultaneous	CI24Re	CP910	29
26	m	Inner ear malformation	Sequential	CI422	CP920	30
27	m	Unknown	Simultaneous	CI512	CP810	31
28	f	Pendred-Syndrome	Simultaneous	HiRes90K Advantage	Harmony	31
29	f	Unknown	Simultaneous	CI422	CP810	39
30	f	Unknown	Simultaneous	CI512	CP810	42
31*	f	Unknown	Simultaneous	CI512	CP810	45
32	m	Unknown	sequential	CI512	CP810	50

After implantation, all children entered the rehabilitation program of our facility. There, they received a bimonthly fitting of the speech processor and multidisciplinary speech and language therapy for up to three years. Age at first activation of the implant ranged from 9 to 50 months (*M* = 20.63 months, *SD* = 11.33 months, *Mdn* = 17 months). Inclusion criteria were bilateral implantation and that in case of a sequential implantation, the second implant was provided within less than a year to avoid adverse affects of asymmetrical input^40,41^ (range of time between sequential implant activations: 1–7 months; median: 3 months). Also, the participants should have no diagnosed disability other than the sensorineural hearing loss when starting with the first measurement.

The EEG recordings were performed 12, 18 and 24 months (+/−2 months) after first implant activation. For each session we obtained written informed consent from the legal guardian of the child. The study was approved by the local ethics committee (Technische Universität Dresden, Germany, IRB00001473) and all methods were performed in accordance with the relevant guidelines and regulations. Sometimes, not all three recordings could be obtained for a child due to occasional illness or excessive restlessness during the session. Final size and distribution of the chronological age within the auditory age groups are listed in Table 2.

**Table 2:** Group size and chronological age in months for the final auditory age groups. In brackets are the respective details of the congenitally deaf children only.

	12 months	18 months	24 months
N	22 (12)	20 (13)	20 (12)
Median age	31.5 (27.5)	35.5 (34)	44.5 (40)
Age range	21–65 (22–58)	29–66 (29–61)	34–76 (34–66)

Strictly, we would have to exclude children from signing families from the evaluation of the congenital group as they had an already established language system in contrast to the congenitally deaf children who had no language access prior to the acoustic input from the implant. Due to the already small numbers in the study and because the numbers of signing children were equally distributed among language performance groups, we refrained from doing so (of the six children with native signing parents, all were congenitally deaf and two were low performers, two norm performers and two high performers).

### Stimuli and procedure

Stimuli and paradigm originate from the study of Friedrich and Friederici^1^ with the exception that no pseudowords or nonwords were presented. The stimuli consisted of 44 words like “apple” or “ball” – considered being among the basic vocabulary developed by one-year-old children – and their matching 44 coloured pictures. The words were spoken in child-directed manner with a mean length of 1083 ms.

Stimuli were presented with the software Presentation® (NeuroBehavioral Systems, Albany, CA). During the passive picture-word-matching paradigm the child would sit 1.5 m away from a computer screen on the lap of the parent, the child’s head level with the screen. During each trial, a picture would appear on the screen for a total of 4000 ms. Following 900 ms after the picture onset, an indefinite article would be presented via loudspeakers, which after an interstimulus interval (ISI) of 1000 ms would be followed by a basic level word. Following the end of the picture presentation, the next picture would be seen on screen without a further ISI and the next trial would begin. The auditory stimuli were presented at 65 dB SPL.

Each session contained two blocks. In one block, half of the pictures were paired with the correct word (“apple” – apple: congruous trial) and half of them with an incorrect word (“apple” – duck: incongruous trial). All items that were incongruously paired in the first block were congruously paired in the second block and vice versa for formerly congruously paired items. Thus, during each session every word item was presented once with a congruous and once with an incongruous picture.

During the session the child’s behaviour was observed closely and only trials where the child was looking at the picture when the word presentation began entered the analysis.

### EEG recording

Data were obtained continuously with Ag-AgCl^−^ electrodes positioned according to the International 10–20 System in an elastic electrode cap (EasyCap, GmbH, Herrsching, Germany). Nine scalp sites (F3, Fz, F4/C3, Cz, C4/P3, Pz, P4) and the left and right mastoid were recorded. An electrooculogram was obtained from two horizontal electrodes at the outer canthi of the left and right eye and from a vertical electrode above the right eye. An additional vertical electrode was recorded below the right eye whenever possible. It was omitted if otherwise the child would not have tolerated the EEG measurement. The signal was sampled at 500 Hz and amplified with a PORTI-32/MREFA (Twente Medical Systems, Oldenzaal, Netherlands) with the electrode Cz as online reference. Impedances were generally kept below 10 kΩ with a few exceptions below 50 kΩ.

Data were rereferenced to the average of both mastoids. If one mastoid was too corrupted by artefacts, the other mastoid served as single reference. A band-pass filter of 0.3–20 Hz reduced slow drifts and muscle artefacts. Trials where the signal exceeded 100 μV within a 200 ms sliding window were rejected. A subsequent correction of eye blinks and eye movements was applied (EEP 3.2.1, developed by the CBS MPI, Leipzig, Germany and distributed by ANT Neuro, Enschede, Netherlands) and all trials were manually re-checked before entering analysis to ensure optimal quality. All sessions had a minimum of 30 trials per condition. Averaging occurred from −200 to 1600 ms with reference to the onset of the basic level word. The 200 ms before stimulus onset served as baseline.

As we analyze the difference between the two conditions and as any potential artefact caused by the CI should be equally present for both congruent and incongruent stimulus pairs, we expect the difference wave to be unaffected. Also, for longer and more complex stimuli the CI artefact tends to be milder^42^, which is what we observed in our data as well.

### Language test

Twenty-four months after first activation of the implant, our participants were tested as a standard procedure with the SETK-2, a German language assessment test^43^. The test is standardised for 24- to 29-month-old children, i.e. normal hearing children with the same amount of auditory experience as our implanted group at the end of our ERP measurements. The test comprises the following four subtests: receptive vocabulary (word level), productive vocabulary (word level), sentence comprehension, and sentence production. The test performance was categorized either as “low” (if performance in at least two subtests was below the norm: t < 40; i.e. in the lower 14% of the norm population)), “norm”, or “high” (if performance in at least two subtests was considered above the norm: t ≥ 60, i.e. better than 82% of the norm population). We chose to include all four subtests in forming the resulting categories rather than just the receptive word vocabulary for reasons of robustness. Performance may vary greatly during the test depending on motivation of the child and thus a single subtest might miss the actual language level of the child.

### Data analysis

Motion artefacts are very common for children that age, but in contrast to the normal hearing population the number of children available for this study is very limited. Analyzing all measured electrodes would have either led to an unequal number of participants per electrode or to a much smaller subset of children being included in the analysis. We thus opted for analyzing only a defined set of electrodes that should nevertheless capture an N400 effect: Cz (in the Supplementary Information) and Pz. The ERP data were averaged within 200ms-long windows starting at 100 ms and ending at 1300 ms after stimulus onset (100–300 ms, 300–500 ms, etc.). In order to test for a significant difference between congruent and incongruent trials, we chose to perform a permutation approach to regression using the lmp function of the R lmPerm package^44^. Permutation tests are recommended for small and possibly non-normally distributed data sets^45^. They thus address the small number of subjects per cell, especially in the subgroup of congenitally deaf children, which result from dividing the age groups into further subgroups according to language performance. In the linear model for the permutation test we defined *amplitude* as the response variable and *condition* (congruent/incongruent) as the predictor variable. For each time window, we performed a permutation test for each age group and each of its language performance subgroups. These tests were repeated for the subgroup of congenitally deaf children. The significance level was set to α = 0.05.

### CI artefact

The amount of trials for each condition was about the same (mean ratio between congruent vs. incongruent trials: 1.00; statistical difference of each dataset’s ratio to the perfect ratio of 1: n.s.). Since we analyze the difference between the conditions and we expect the artefact to be present in both conditions, any potential artefact should be minimal or eliminated. This was confirmed by visual data inspection of the difference wave between the two conditions.

### Data availability

The data that support the findings of this study are available in non-identifiable format from the corresponding author upon reasonable request.

## Results

We compared the ERPs of the non-congenitally deaf children to the ERPs of the congenitally deaf children and found no statistical difference between them in any age and/or performance group. We still report the results of the congenitally deaf children additionally, since they form a particularly interesting group of individuals whose encounter with oral language has occurred exclusively with the CI in contrast to children who had some prior hearing before implantation and whose results may thus be influenced by some initial language exposure. Please note though, that participant numbers are low and need to be treated with the appropriate caution.

### Language test results

Of the 32 children, 13 (41%) performed above the norm range in at least two subtests (high performers, 8 congenitally deaf), 9 (28%) performed below the norm range in at least two subtests (low performers, 7 congenitally deaf), and the other 10 (31%) children performed within the norm range or had only one test deviating positively or negatively (norm performers, 4 congenitally deaf). Numbers and chronological age per auditory age group are summarized in Table 3. No child falling into the category of high performers performed below the norm range in any of the subtests and vice versa for the low performers.

**Table 3:** Group size and chronological age of all cochlear implanted children and the subgroup of congenitally deaf children per age group and per language performance group.

all						congenital					
		12 months	18 months	24 months	Total			12 months	18 months	24 months	Total
**low**	N	6	5	8	19	**low**	N	4	3	5	12
	median	31.5	42	44.5	38		median	27	34	38	34
	age range	22–53	30–57	34–65	22–65		age range	22–37	30–42	34–47	22–47
**norm**	N	8	8	5	21	**norm**	N	4	5	2	11
	median	38.5	35.5	54	44		median	32.5	35	55.5	35
	age range	21–64	30–66	38–76	21–76		age range	22–58	30–61	46–65	22–65
**high**	N	8	7	7	22	**high**	N	4	5	5	14
	median	25.5	32	37	33.5		median	25.5	32	37	33
	age range	21–52	29–46	33–54	21–54		age range	21–30	29–46	34–38	21–48

Note that test data were unavailable for 3 of the 32 children. Language test results 24 months post-implantation were missing for one child. Speech therapists evaluated her language development as being extremely good and the child was categorized as high performing. For the other two children, language development at 24 months post-implantation did evidently not meet the requirements for performing the SETK-2 test, so that no test was attempted. These children were automatically categorized as “low performers”.

### ERP results

**Figure 1:**
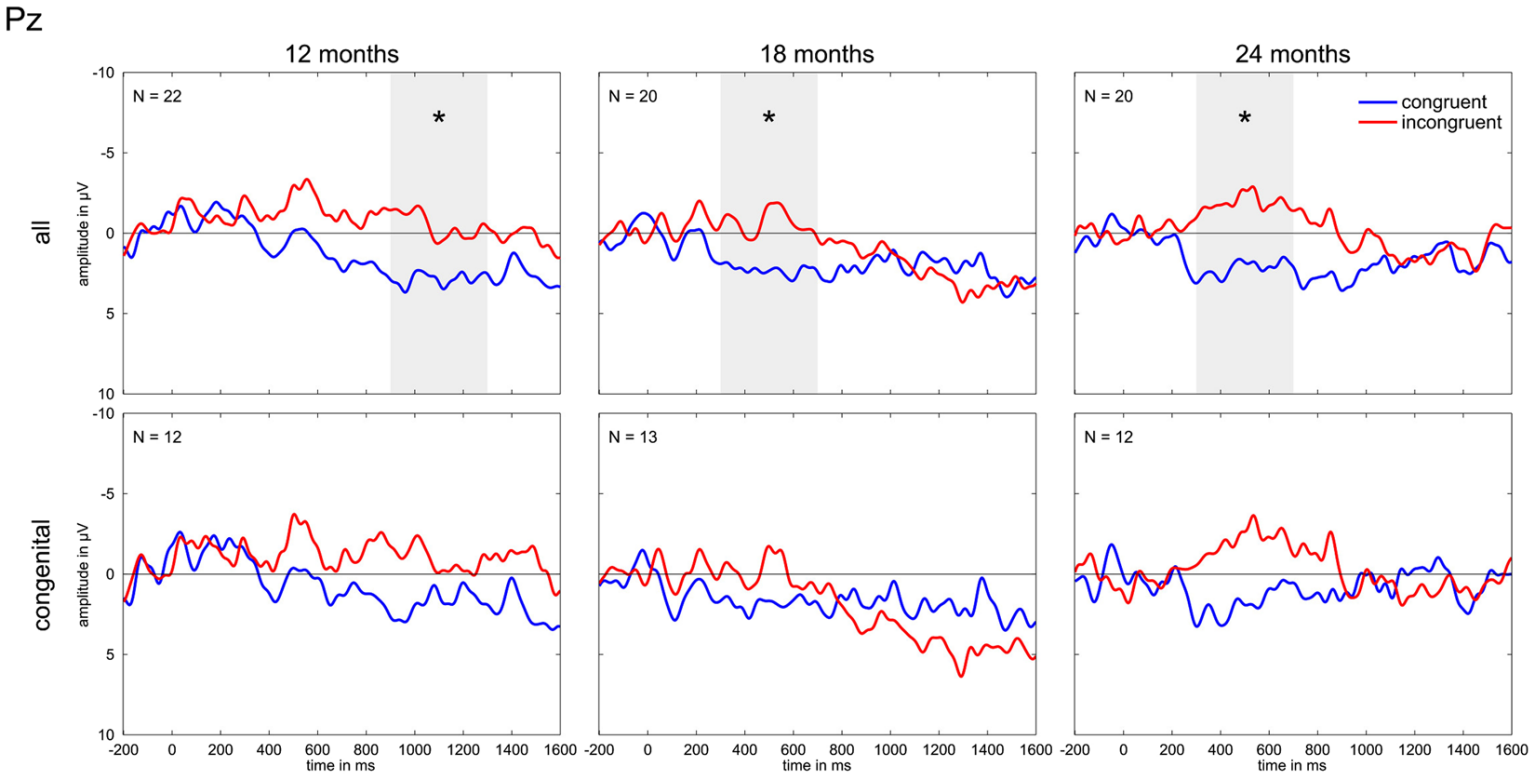
ERPs at electrode Pz at 12, 18 and 24 months. The second row represents the subgroup of congenitally deaf children. Time windows with a significant difference between the two conditions (p < 0.05) are marked by a grey area and an asterisk.

Figure 1 displays the ERP data split by hearing age at electrode Pz, Table 4 summarizes the statistical results (see Supplementary Fig. S1 and Table S1 for corroborating results of electrode Cz).

**Table 4:** Statistical results of the permutation test. Results for the three age groups and for the language performance groups of each age group at electrode Pz. p < .05.

		**12 months**						**18 months**						**24 months**					
		**100–300 ms**	**300–500 ms**	**500–700 ms**	**700–900 ms**	**900–1100 ms**	**1100–1300 ms**	**100–300 ms**	**300–500 ms**	**500–700 ms**	**700–900 ms**	**900–1100 ms**	**1100–1300 ms**	**100–300 ms**	**300–500 ms**	**500–700 ms**	**700–900 ms**	**900–1100 ms**	**1100–1300 ms**
	**all**					0.011	0.021		0.031	0.032					0.031	0.035			
	**congenital**																		
**all**	**low**																		
	**norm**					0.010	0.007												
	**high**					0.046									0.017	0.047	0.049		
	**norm+high**		0.028	0.020	0.011	0.001	0.045		0.030						0.001	0.007	0.011		
**congenital**	**low**																		
	**norm**					0.048													
	**high**														0.043	0.036			
	**norm+high**					0.020									0.032	0.037			

#### 12 *months*

Already after 12 months of hearing experience with the implant the incongruent pairs displayed an increased negative potential that is significant at late time windows. For the subgroup of congenitally deaf children no incongruity effect was seen.

#### 18 *months*

After 18 months of hearing experience the negativity of the incongruent trials diminished in amplitude, size, and breadth, but was nevertheless statistically significantly more negative than congruent trials. No incongruity effect was found in the subgroup of congenitally deaf children. The tentative significance in the time window 1100–1300 ms is due to the incongruent condition being more positive than the congruent condition.

#### 24 *months*

After 24 months of hearing experience with the CI the ERP course for the incongruent trials was distinctly more negative than for the congruent trials for both the whole group and the subgroup of congenitally deaf children, though for the congenitally deaf children the condition difference did not reach significance except for a very tentative significance in the time window 500–700 ms.

In summary we can conclude that an N400-like effect was visible in all age groups throughout the second year of implant use. The effect does not seem to be driven by prior hearing experience as we saw the same pattern also for the congenitally deaf children alone and there was no statistically significant difference to the children with prior hearing experience. However, probably due to lower power in the congenitally deaf group, the N400 effect was only robustly present in the overall group.

### ERP results of the low, norm, and high performers

**Figure 2:**
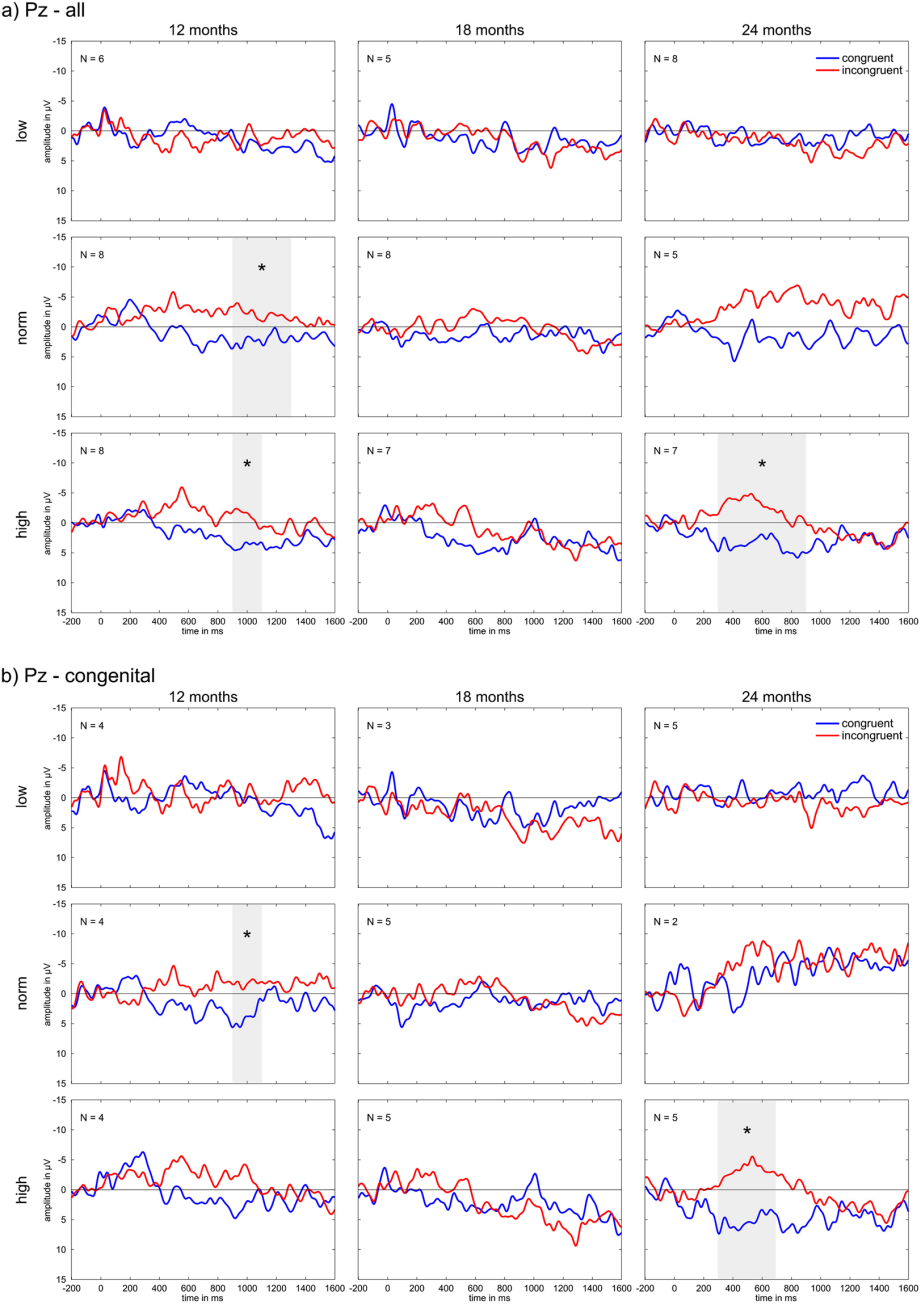
ERPs within the different age groups sorted according to low/norm/high language test performance at electrode Pz for (**a**) all children and (**b**) the congenitally deaf children. Time windows with a significant difference between the two conditions (*p* < 0.05) are marked by a grey area and an asterisk.

We further examined whether there are any developmental differences to be seen when the participants are retrospectively grouped according to their language test results performed at the age of 24 months. We indeed found substantial differences: The group of children that had test results below the norm range (“low performers”) showed practically no difference between the congruent and the incongruent condition throughout the entire second year of implantation. By contrast, those children who reached results within (“norm performers”) and above (“high performers”) the norm range demonstrated a progressively developing N400 effect (see Fig. 2). Encouragingly, the congenitally deaf children were not overrepresented in the low performing group but were instead equally distributed over the three performance groups. The statistical results are summarized in Table 4.

#### Low performers

Children belonging to the group of low performers did not show any significant difference between congruous and incongruous trials at any age group.

#### Norm performers

Children performing within the norm range of the language test displayed a significant difference between the conditions already 12 months after implant activation in the time window 900–1300 ms. For the congenitally deaf children at 12 months, a significant condition effect was visible at 900–1100 ms.

#### High performers

Twelve months after implant activation, the high performing group displayed a significant condition effect in the time window 900–1100 ms. After 24 months, the condition effect became significant at 300–900 ms. For the congenitally deaf children, the condition effect reached significance after 24 months at 300–700 ms.

#### Norm and high performers

**Figure 3:**
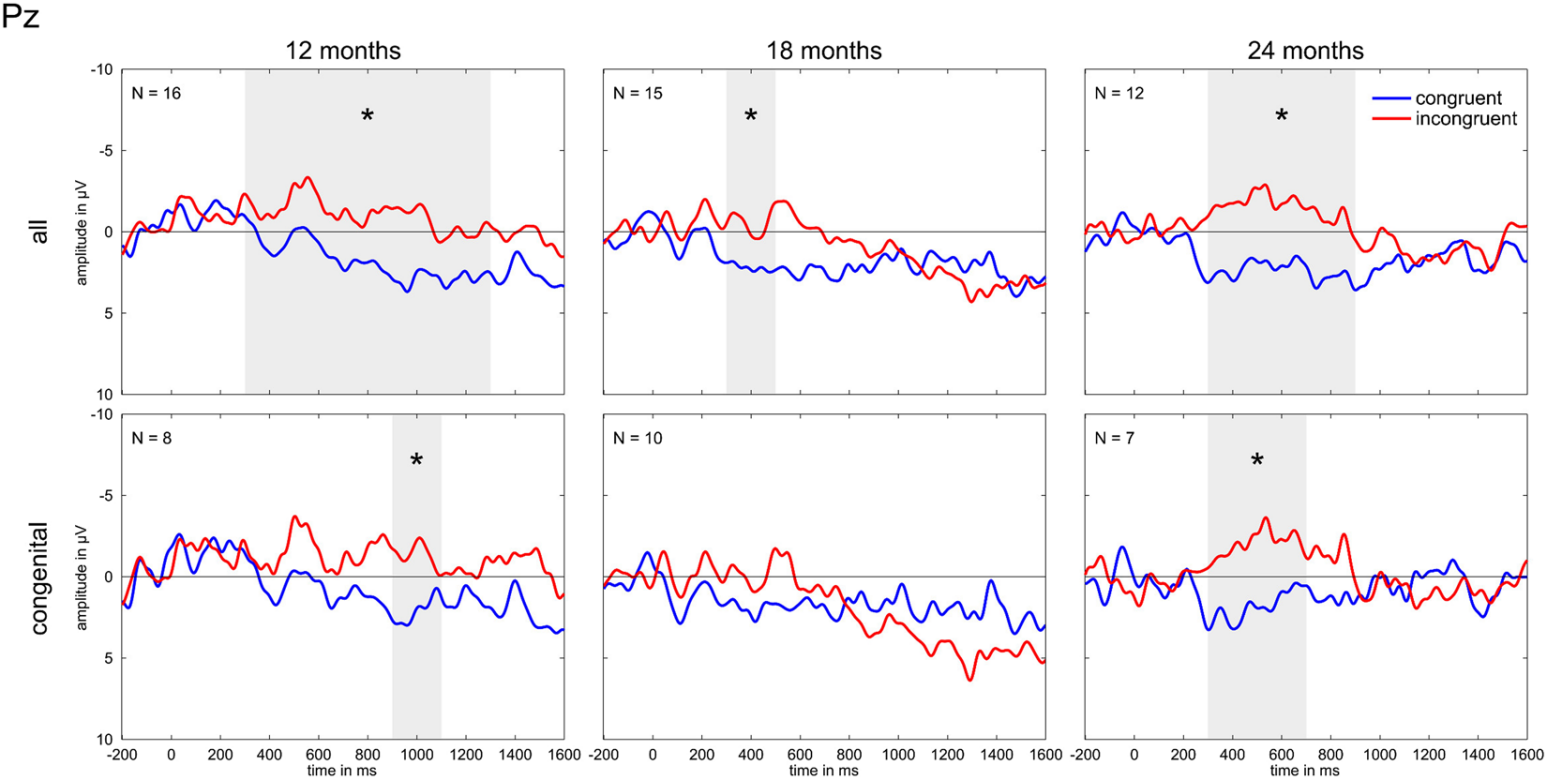
ERPs at electrode Pz averaged over high and norm performers. Top row: all children. Second row: congenitally deaf children only. Time windows with a significant difference between the two conditions (p < 0.05) are marked by a grey area and an asterisk.

As the numbers in the individual groups were rather small – especially for the congenitally deaf subgroups – we performed an additional analysis for the norm and high performers as one group (included in Table 4) in order to see whether the pattern of the norm and high performing group becomes more robust with an increased N. Both groups had a rather similar pattern of ERP development, clearly deviating from that of the low performers and thus justifying the merging of the two better performing groups. The resulting ERPs are depicted in Fig. 3. For the 12-month age group, we found that the significant difference between the conditions in the whole group was surprisingly broad – spanning the time windows from 300–1300 ms – while for the congenitally deaf children this was confined to the 900–1100 ms time window. With both groups taken together, the 18-month-group then also displayed a significant difference in the time window 300–500 ms, but not the congenitally deaf subgroup. The 24-month-group, in turn, showed a significant difference that spanned 300–900 ms in the overall group and 300–700 ms in the subgroup of congenitally deaf children.

### Differences between performance groups

After 12 months of implant use we find that both high and norm performers have a more negative difference wave than the low performers in the time window 500–700 ms (p = 0.013 and p = 0.008, respectively). In addition, the norm performers have a more negative difference wave than the low performers in the time window 300–500 ms (p = 0.048). No statistical difference between performance groups can be found in the auditory age group of 18 months. In the 24 month group, again both high and norm performers have an increased negative difference wave compared to the low performers, this time at 300–500 ms (p = 0.033 and p = 0.028, respectively). When comparing norm and high performers as one group against the low performers, the picture remains the same but with even smaller p-values (12 months: p = 0.045 at 300–500 ms and p = 0.002 at 500–700 ms; 18 months: n.s.; 24 months: p = 0.01at 300–500 ms).

As the low performers differed so clearly from the norm and high performing children in showing no trace of a developing N400 effect even after two years of implant use, we took a closer look at the aetiology and non-verbal cognitive performance of this group. An inclusion requirement was no diagnosed disability when entering the study. However, in the group of low performers seven of the nine children developed signs of an additional impairment either over the course of the study or afterwards. Child 3 has shown developmental delays of >6 months in the motor and cognitive domain in the Bayley Scales of Infant Development II^46^. Child 5 has in the meantime been diagnosed with a pervasive developmental delay. Child 22 is suspected to have a specific phonological memory impairment with otherwise normal cognitive abilities (non-verbal IQ test in the SON-R: 115) and was recently diagnosed with childhood absence epilepsy. Child 21 is without a diagnosis, but has shown a language delay that is beyond the normal late talker phenomenon, while displaying a normal to good IQ of 110 in non-verbal SON-R intelligence test. Child 10 has shown a very slow language progress and an IQ of 51 in the SON-R. Child 16 also displayed a low IQ of 63 in the SON-R. The cause of deafness in child 25 was meningitis, which is known for potentially affecting cognitive abilities. No SON-R scores were available for this child, but the Bayley Scales of Infant Development attested a developmental delay in all categories by about one year. For the other two children there were no signs that could account for a low language performance. One of them (child 29) was implanted rather late (3;3 years) but other children (e.g. child 31 and child 32) were implanted even later and still performed within the norm range. No signs of additional disabilities manifested in children of the norm and high performing groups.

### Effects of maturation and auditory/language experience

Though the ERPs of the low performing children do not show any development towards an N400, the data of the norm and high performing children show an increase of the N400 effect. In order to assess this developmental change statistically, we compared the ERPs of norm and high performing children who were recorded both at the auditory age of 12 and of 24 months (N = 8; of which four congenitally deaf) by means of a permutation test on the difference wave with auditory age as a between-group factor. We found significant differences between the two auditory age groups in the time windows 100–300 ms and 300–500 ms (p = 0.004 and p = 0.032, respectively), which is due to the N400 effect appearing earlier at the auditory age of 24 months than at the auditory age of 12 months. In the first time window, the 12 M group shows a positive difference wave (3.68 µV), while the 24 M group already has a negative difference wave (−3.13 µV). In the time window 300–500 ms, the 12 M group has a difference wave close to zero (0.09 µV), while the 24 M group has a large difference wave value indicative of an N400 effect (−7.87 µV). In contrast, there is no difference of amplitude in the later time windows. The maturation from 12 to 24 months is thus reflected in a latency shift and not in an increase of amplitude.

### Age at implantation

A correlation of age at implantation and the amplitude of the N400 effect with age as a continuous variable yielded no statistically significant results. Only when performing a comparison between early and late implanted children by placing the cut-off age at 18 months – but not when setting the cut-off age at 12 months or 24 months – we find a significant relationship: Norm and high performing children implanted before or at the age of 18 months (N = 6) have a larger N400 effect in the time window of 500–700 ms after 18 months of hearing experience (−6.67 µV) compared to children implanted at an earlier age (N = 6; 0.042 µV; p = 0.037).

## Discussion

The aim of this study was to assess the lexical-semantic development of profoundly hearing impaired and deaf children with a cochlear implant using the N400 effect as its marker. We investigated the period of the second year of implant use as this is the auditory age at which in NH children vocabulary emerges and undergoes the so called vocabulary spurt.

Consistent with our initial hypothesis, we found that cochlear implanted children show a typical N400 effect developing throughout the second year of auditory experience with cochlear implants. That is, an incongruent picture-word pair elicited a larger negative deflection in the EEG than a congruent pair already after 12 months of implant use. The latency of the N400 effect shortened over time and after 24 months of implant use the ERP strongly resembled that of normal hearing children of a similar auditory age^47,48^. This provides evidence for robust semantic relationships between words and objects having formed already after one year of implant use. Though we did not expect the ERP amplitude to diminish at 18 months, it is consistent with similar studies from normal hearing children of the same auditory age, where 19-month-old children displayed a smaller amplitude than 14-month-old children^49^. The fact that the N400 effect is stronger and earlier at parietal electrode sites over all age groups is also consistent with studies of normal hearing children of the same auditory age^16,47,48^ and was likewise seen in older implanted children^39^.

In addition, we conducted a retrospective grouping of the children based on language test results 24 months after implant activation as we hypothesized a different ERP development for high vs. low performing individuals. We found indeed such a difference that was even larger than expected. Children whose performance fell within or above the norm range displayed a developing N400 effect starting as early as 12 months after implantation and reaching the latency and distribution of normal hearing auditory age peers by 24 months^50^. The ERPs obtained at 24 months also resembled those displayed by much older 5- to 7-year-old implanted children with several years of implant experience^39^.

In striking contrast to the norm and high performers, the low performers did not show any hint of an N400 effect in either age group, neither statistically nor descriptively. For a large proportion of these children, the missing sign of developing semantic relationships might be explained by the respective additional disability. For the other children, we may speculate whether it is (a) poor auditory input that leads to poor language development, either due to the quality of the input or due to unfortunate consequences of the period without any auditory input, or (b) whether this is within the normal range of language development. For the latter hypothesis speaks that in studies with NH children, low performers without language impairments also failed to display an N400 at 19 months^47^, and they equally failed to display an N400 for newly learned words at the age of 20 months as opposed to high performing children^51^. By contrast, if the implanted low performing children continue not displaying an N400 effect at an older age, insufficient auditory input or processing is the more likely reason. We know from children with specific language impairment who are suspected to have problems with auditory processing that semantic irregularities within sentence context do not elicit an N400, even at the age of 9–10 years^52^. We thus must await future language and ERP assessments of the low performing CI children to better understand the underlying cause.

The subgroup of the congenitally deaf children is always of particular interest as they acquire language purely based on input provided by the implants. For these children, any input-dependent maturation of auditory processes starts no earlier than at first implant activation. Indeed, at first glance it would seem that the congenitally deaf children did not display a significant N400 effect, though it was present descriptively. A closer look, however, revealed a similar pattern as seen for the overall group: While the low performing individuals of the congenitally deaf group showed no trace of semantic processing, the norm and high performing individuals did demonstrate a significant N400 effect. Even more encouraging, and contrary to our hypothesis that expected a slower development for the congenitally deaf group than the overall group, the effect was also already present after 12 months of implant use and thus earlier than for NH children with the same amount of language exposure^53^.

Congenitally deaf children with a CI provide a unique model of children who encounter spoken language for the first time when other cognitive faculties have already matured to a certain degree. These children can thereby help to understand the interdependencies of language acquisition and the maturation of other cognitive domains. Friedrich and Friederici^15^, for example, showed that though 6-month-old infants display an N400 after being trained on word-picture pairs, that effect vanishes the next day. They argue that 6-month-old infants are already capable of semantic encoding but that a true vocabulary depends on a more mature declarative memory that would ensure the retention of the learned word-object pairs. Considering that nowadays congenitally deaf children are implanted on average at the chronological age of 12 to 24 months, they should already have the general memory structures for retaining newly learned words by the time they are immersed in oral language^54^.

In addition, children not immersed in a structured language (oral or signing), will nevertheless derive some categories based on certain properties (visual and other) of their everyday environment and we would expect semantic language development to capitalize on this prior knowledge as soon as language access is provided. Infants start forming spatial concepts at the age of 6 months before relating them to language^55^ and can be trained on semantic categories by the age of 6–7 months^56–58^. Also, extensive research by Linda Smith and colleagues has shown that increased attention (e.g. to specific visual properties) and category formation affect word acquisition: Being able to attend to specific details like object shape and being able to generalize from shape to a higher-order category positively correlates with productive vocabulary^59^. Training on certain visual features like shape increases vocabulary acquisition even outside the lab^60^, while the reverse is also true. “Late talkers”, i.e. children with an unusually small productive vocabulary for their age have difficulties in recognizing abstract shapes, whereas younger children who have the same size of vocabulary perform better^61,62^.

The benefit of matured cognitive faculties for word learning might also explain why we found only a small advantage for children implanted before the age of 18 months compared to children implanted later, though the effect was limited to one time window and only showed after 18 months of implant experience and should be treated with caution. The overall effect of maturation is also likely to be restricted to basic word learning and not generalizable to other language aspects that might be more severely affected by a delayed first-language onset^63^.

One limitation of the study is the relatively small number of implanted children once they are divided into language performance groups, especially when subdivided again into congenitally deaf groups vs. the overall group. Given that the yearly implantations that follow our inclusion criteria (bilaterally implanted, no further diagnosed disability at the beginning of the study, age range) are limited in numbers, it will take considerable time to markedly increase the number of participants. This would, however, be highly valuable in order to confirm the present results and in order to form more precise age groups.

We conclude that not only the high performing children, but indeed the majority of implanted children display a development very similar to NH children: For both norm and high performers an N400 effect develops alongside the behavioural emergence of vocabulary, starting 12 months after being immersed in spoken language. Thus, despite the fact that children with a CI deal with more adverse listening situations than their NH peers, the majority of implanted children demonstrate a semantic development at least parallel to that of normal hearing children of the same auditory age. The ERP results confirm the validity of the SEKT-2 language test in that the low performance group indeed lag substantially behind in language acquisition. The ERPs, however, reveal that at risk children diverge from normal and high performing children already at the auditory age of 12 months and thus detect them one year earlier than the language test. Also, the ERP results suggest that the difference between the low performing and the better performing children is not of a gradual, quantitative nature, but rather a qualitative one, as the N400 effect appears not smaller or later, but is altogether absent throughout the entire second year of implant use. This is especially worrisome as the chronological age of the implanted children is higher than their auditory age. This might not be a problem for later word acquisition as some studies suggest that the learning of single words has no strict developmental time limit. However, the learning of grammatical rules seems to be more sensitive to developmental time frames^63,64^ and a missing mental lexicon might further constrain complex language acquisition.

In the present study we have shown that implanted children display an N400 effect at the auditory age of 12 months, which is slightly earlier than for normal hearing children – possibly due to their higher chronological age. The next step would be to test whether congenitally deaf children show an N400 effect at an even earlier auditory age, or whether the nature of the CI input or other factors underlying language acquisition are setting limits to how fast language can actually be acquired.

## Electronic supplementary material

Supplementary material
