# Supplementary material for: Establishing a mental lexicon with cochlear implants: an ERP study with young children
Source: Sci Rep. 2018 Jan 17;8:910. doi: 10.1038/s41598-017-18852-3 (PMC5772553; doi:10.1038/s41598-017-18852-3)
Supplement: Supplementary file 1 — Supplementary material [file 41598_2017_18852_MOESM1_ESM.doc]

# Establishing a mental lexicon with cochlear implants: an ERP study with young children

Niki K. Vavatzanidis ^1,2^*, Dirk Mürbe ^2^, Angela D. Friederici ^1^, Anja Hahne ^2^

^1^ Max Planck Institute for Human and Cognitive Brain Sciences, Leipzig, Germany

^2^ Saxonian Cochlear Implant Center, Technische Universität Dresden, Dresden, Germany

Contact information: vavatzanidis@cbs.mpg.de


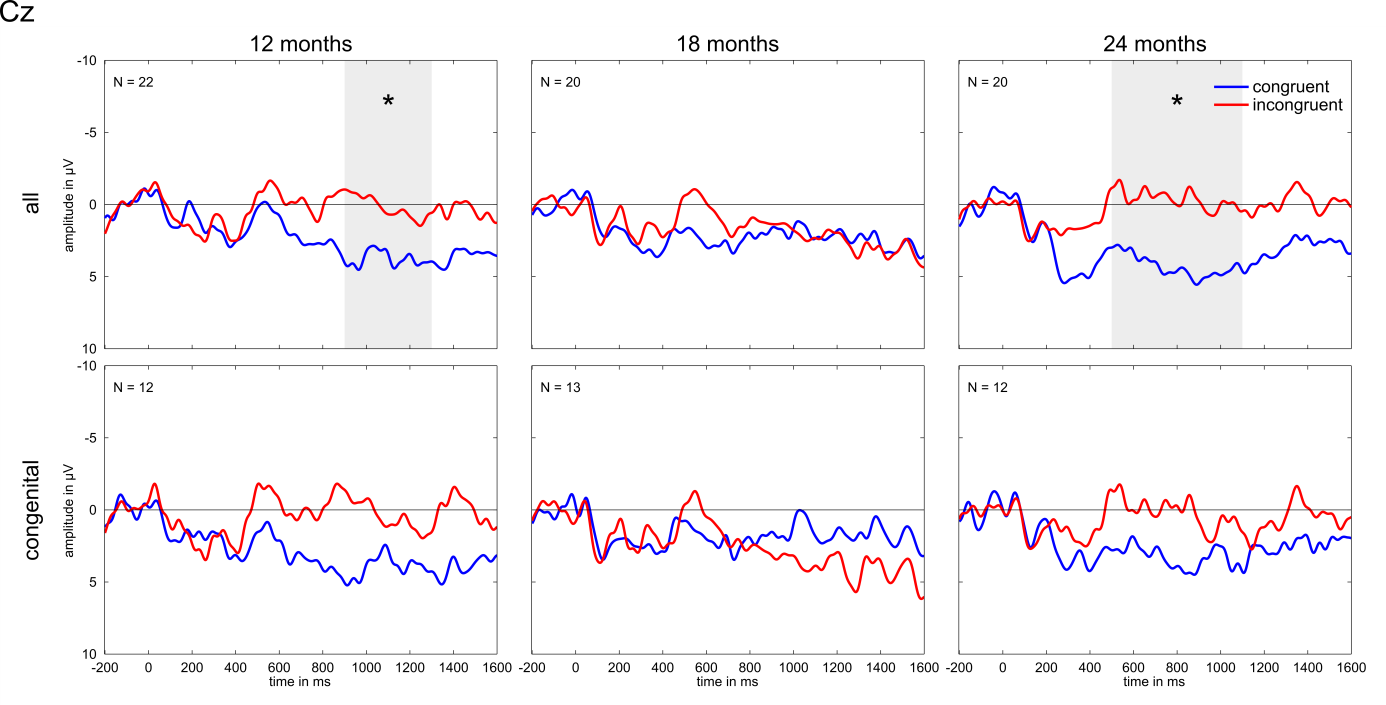


Figure S1: ERPs at electrode Cz at 12, 18 and 24 months. The second row represents the subgroup of congenitally deaf children.

Table S1: Statistical results of the permutation test. Results for the three age groups and for the language performance groups of each age group at electrode Cz. p < .05

|  | | | **12 months** | | | | | | **18 months** | | | | | | **24 months** | | | | | |
| --- | --- | --- | --- | --- | --- | --- | --- | --- | --- | --- | --- | --- | --- | --- | --- | --- | --- | --- | --- | --- |
|  |  |  | 100-300ms | 300-500ms | 500-700ms | 700-900ms | 900-1100ms | 1100-1300ms | 100-300ms | 300-500ms | 500-700ms | 700-900ms | 900-1100ms | 1100-1300ms | 100-300ms | 300-500ms | 500-700ms | 700-900ms | 900-1100ms | 1100-1300ms |
| **Cz** | **all** | |  |  |  |  | 0.009 | 0.019 |  |  |  |  |  |  |  |  | 0.041 | 0.018 | 0.031 |  |
|  | **congenital** | |  |  |  |  |  |  |  |  |  |  |  |  |  |  |  |  |  |  |
|  | **all** | **low** |  |  |  |  |  |  |  |  |  |  |  |  |  |  |  |  |  |  |
|  |  | **norm** |  |  |  |  |  |  |  |  |  |  |  |  |  |  |  | 0.037 |  | 0.042 |
|  |  | **high** |  |  |  |  | 0.037 |  |  |  |  |  |  |  |  |  |  | 0.026 |  |  |
|  |  | **norm+high** |  |  |  |  | 0.008 |  |  |  |  |  |  |  |  | 0.021 | 0.013 | 0.003 | 0.009 | 0.011 |
|  | **congenital** | **low** |  |  |  |  |  |  |  |  |  |  |  |  |  |  |  |  |  |  |
|  |  | **norm** |  |  |  |  |  |  |  |  |  |  |  |  |  |  |  |  |  |  |
|  |  | **high** |  |  |  |  |  |  |  |  |  |  |  |  |  |  |  | 0.044 |  |  |
|  |  | **norm+high** |  |  |  |  | 0.042 |  |  |  |  |  |  |  |  |  | 0.044 | 0.027 |  |  |


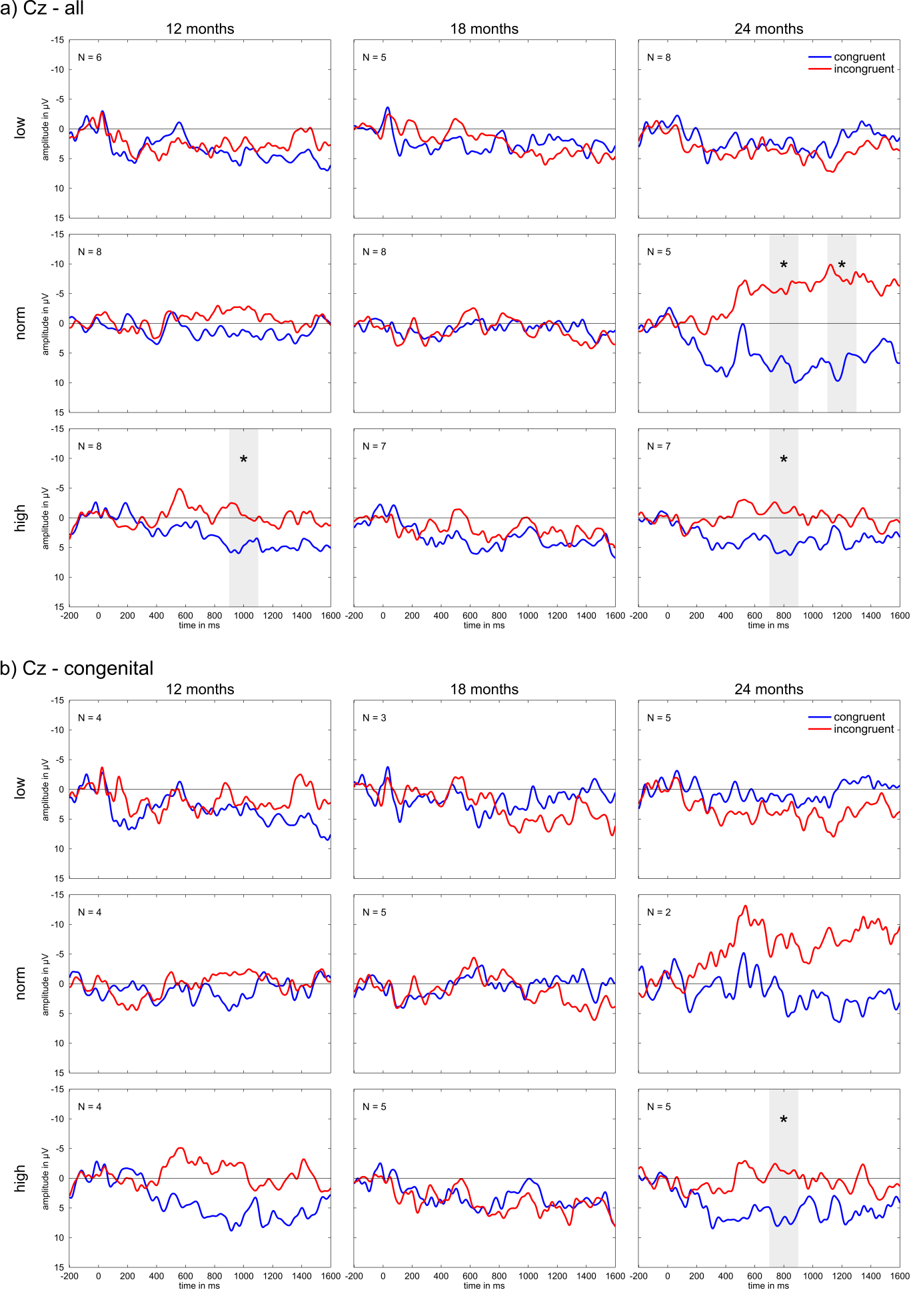


Figure S2: ERPs within the different age groups sorted according to low/norm/high language test performance at electrode Cz for a) all children and b) the congenitally deaf children.


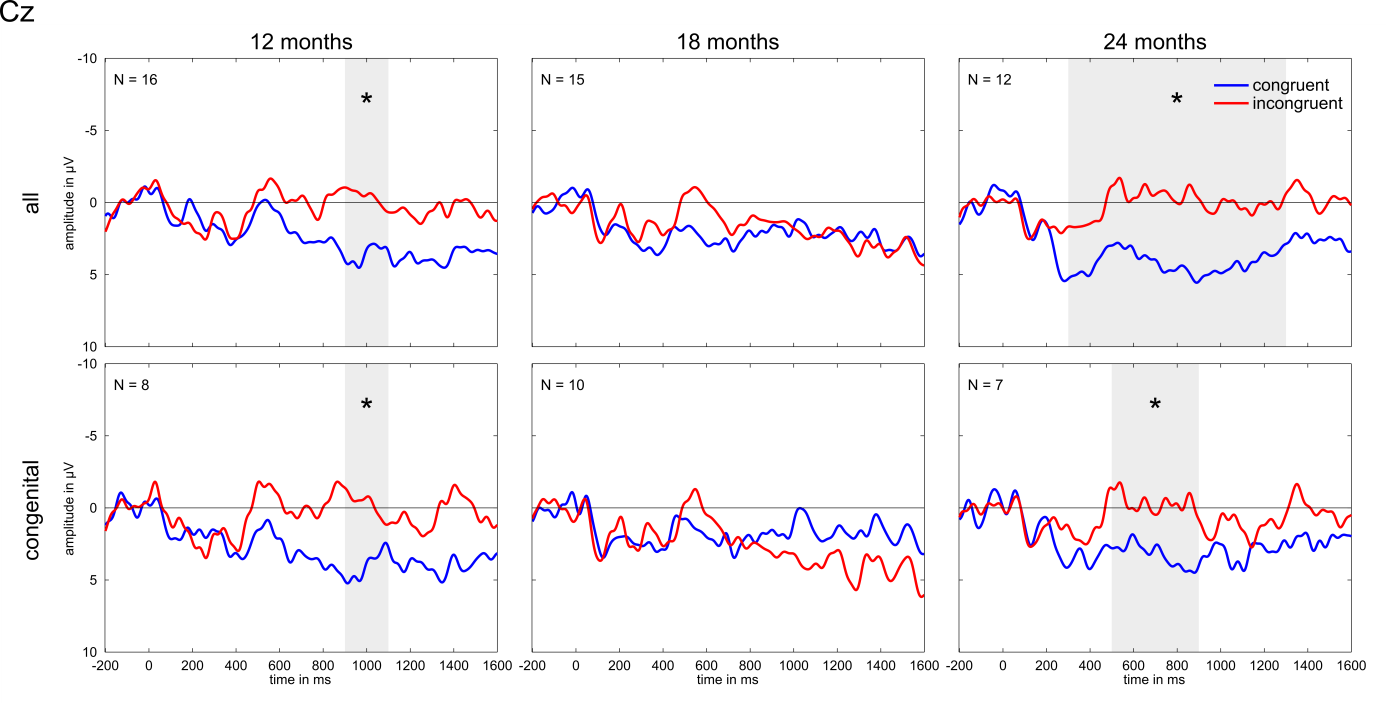


Figure S3: ERPs at electrode Cz averaged over high and norm performers. Top row: all children. Second row: congenitally deaf children only.

Effects of maturation and auditory/language experience

At Cz the significant difference between the two auditory age groups appeared also in the time windows 100-300 ms and 300-500 ms (p = .015 and p = .017, respectively). In the first time window, the 12M group shows a positive difference wave (3.81 µV), while the 24M group already has a negative difference wave (-3.87 µV). In the time window 300-500 ms, the 12M group has still a positive difference wave (2.04 µV), while the 24M group has a large difference wave value indicative of an N400 effect (-7.57 µV). Like at Pz, there is no difference of amplitude in the later time windows suggesting that maturation from 12 to 24 months is reflected in an earlier latency rather than an increase of amplitude.

Age at implantation

When placing the cut-off age at 12 months, we find for the norm and high performing children a significant difference (p = .027) between the earlier implanted children (N = 3; -7.94 µV) and the later implanted children (N = 9; 0.11 µV) after 12 months of hearing experience in the time window 500-700 ms.

Differences between performance groups

At Cz, the performance groups do not differ in their difference wave after 12 and 18 months of implant use. Instead we find more time windows with group differences than at Pz at 24 months of implant use. The low performance group differs in every window of analysis from the norm performance group (100-300 ms: p = .049; 300-500 ms: p = .009; 500-700ms: p = .011; 700-900 ms: p = .004; 900-1100 ms: .002; 1100-1300 ms: p < .001). Interestingly, the norm performance have an even more negative difference wave than the high performing group (300-500 ms: p = .03; 500-700 ms: p = .048; 700-900 ms: p = .036; 900-1100 ms: p = .014; 1100-1300 ms: p = .007), while the high performing group does not differ significantly in direct comparison to the low performing group, though it is marginally significant in two time windows (300-500 ms: p = .076; 700-900 ms: p = .085). However, when pooling the high and norm performing group together, the resulting p-values are lower than for the norm performing group alone (300-500 ms: p = .012; 500-700 ms: p = .009; 700-900 ms: p = .004; 900-1100 ms: p = .026; 1100-1300 ms: p = .016). This analysis tests only if the difference wave between the groups is significantly different, not whether the difference wave is per se significantly different from zero within the groups (i.e. whether the conditions differ significantly).
